# Enzyme Inhibitory Radicinol Derivative from Endophytic fungus *Bipolaris sorokiniana* LK12, Associated with *Rhazya stricta*

**DOI:** 10.3390/molecules200712198

**Published:** 2015-07-03

**Authors:** Abdul Latif Khan, Liaqat Ali, Javid Hussain, Tania Shamim Rizvi, Ahmed Al-Harrasi, In-Jung Lee

**Affiliations:** 1 UoN Chair of Oman’s Medicinal Plants and Marine Natural Products, University of Nizwa, Birkat Al-Mouz, Nizwa 616, Oman; E-Mails: abdullatif@unizwa.edu.om (A.L.K); javidhej@unizwa.edu.om (J.H.); tania@unizwa.edu.om (T.S.R.); 2 Department of Biological Sciences and Chemistry, College of Arts and Sciences, University of Nizwa, Birkat Al-Mouz, Nizwa 616, Oman; 3 School of Applied Biosciences, Kyungpook National University, Daegu 702-701, Korea; E-Mail: ijlee@knu.ac.kr

**Keywords:** radicinol, bipolarisenol, *Rhazya stricta*, *Bipolaris sorokiniana* LK12, lipid peroxidation, enzyme inhibition

## Abstract

Endophytes, living inside plant tissues, play an essential role in plant growth and development, whilst producing unique bioactive secondary metabolites. In the current study, the endophytic fungus *Bipolaris sorokiniana* LK12 was isolated from the leaves of ethno-medicinal and alkaloidal rich *Rhazya stricta*. The bulk amount of ethyl acetate extract of fungus was subjected to advance column chromatographic techniques, which resulted in the isolation of a new radicinol derivative, bipolarisenol (**1**). It was found to be a derivative of radicinol. The structure elucidation was carried out by the combined use of 1D and 2D nuclear magnetic resonance, infrared spectroscopy, mass, and UV spectrometric analyses. The bipolarisenol was assessed for its potential role in enzyme inhibition of urease and acetyl cholinesterase (AChE). Results showed that bipolarisenol significantly inhibited the AChE activity with low IC_50_ (67.23 ± 5.12 µg·mL^−1^). Bipolarisenol inhibited urease in a dose-dependent manner with high IC_50_ (81.62 ± 4.61 µg·mL^−1^). The new compound also showed a moderate anti-lipid peroxidation potential (IC_50_ = 168.91 ± 4.23 µg·mL^−1^). In conclusion, endophytes isolated from medicinal plants possess a unique potential to be considered for future drug discovery.

## 1. Introduction

Endophytic microbes (bacteria and fungi) live inside plant tissues without causing any symptoms of disease to the host. Endophytic fungal association extends benefits to the plants by changing the endogenous mineral nutrients and metabolites levels [1]. During symbiosis, these endophytes produce metabolites inside plant tissues to help in the improvement of plant defenses against environmental stresses [2,3,4,5]. A significant number of interesting molecules have been produced by endophytes, including flavonoids, peptides, alkaloids, steroids, phenolics, terpenoids, lignans, and volatile organic compounds with many of them biologically active [5,6]. In fact, a previous study by Schulz *et al.* [6] showed that about 51% of biologically active metabolites originate from endophytes compared to only 38% of novel substances originating from other soil microflora. In the past two decades, many novel bioactive compounds with antimicrobial, insecticidal, cytotoxic, and anticancer properties have been successfully isolated and characterized from endophytic fungi [4,7].

One of the interesting examples of endophytic bioactive secondary metabolites is the production of the world’s first billion dollar anticancer drug, paclitexel (taxol). Although it was initially reported from *Taxus brevifolia*, later it was also isolated from various endophytes (*Taxomyces andreanae*, *Botryodiplodia theobromae*, and *Pestalotiopsis microspore* *etc.*) living in symbiosis with the host [8]. The endophyte colonizes the Himalayan yew tree without causing apparent injury to the host plant. This potential has set the stage for increasing interest in fungal endophytes [3,9]. Looking at this potential, in the current study *Rhazya stricta* Decne was selected for its endophytic microbes and their potential to produce bioactive metabolites. *R. stricta* grows wildly in various countries of South Asia and the Middle East. It is a medicinal plant largely known for its alkaloidal content [10]. More than 100 different alkaloids have been reported from this plant [11,12]. Some of the chemical constituents have a potent function in various pharmacological activities.

**Figure 1:**
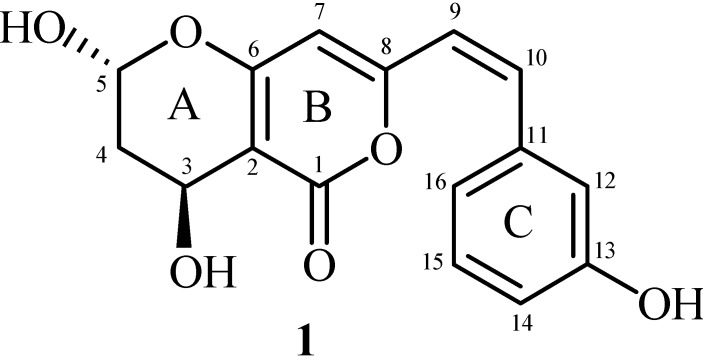
Structure of the new radicinol derivative, bipolarisenol (**1**).

Since the host *R. stricta* was found rich in diverse kinds of secondary metabolites, we aimed to explore its symbiotic endophytic fungi for similar potentials. The endophytic fungal diversity is yet to be explored from *R. stricta*. In the current study, a dark septet endophytic fungus, *Bipolaris sorokiniana LK12* was isolated for the first time from the leaf part of *R. stricta*. The metabolites produced at extracellular level during the growth of *B. sorokiniana* were assessed using advanced chromatographic and NMR spectroscopic techniques. As a result, one new secondary metabolite belonging to radicinol class, bipolarisenol (**1**) (Figure 1), was purified and the chemical structure was elucidated. Furthermore, the purified constituent was subjected to biological assays to access the medicinal potential of the metabolite from endophyte *B. sorokiniana.*

## 2. Results and Discussion

The ethyl acetate extract obtained from the pure culture filtrate of endophytic fungus was assayed for possible bioactive role. Initial screening results showed that the extract (100 ppm) possessed significantly higher anti-lipid peroxidation potential (51.28% ± 0.97%). Further fractionation of the ethyl acetate extract resulted in the isolation of compound **1**. Compound **1** was isolated as a colorless amorphous solid and the structure elucidation was carried out by the analysis of 1D and 2D NMR, MS, and IR spectral data along with comparison of the related NMR and MS data in literature [13,14].

The IR spectrum of compound **1** exhibited absorption bands at 3595 (OH), 1695 (C=O), and 1590 cm^−1^ (C=C). The presence of hydroxyl groups was indicated by the IR band at 3595 cm^−1^. The UV maxima at 340 and 264 nm indicated the presence of conjugated aromatic system in the molecule [15]. The analysis of ESI-MS indicated the presence of pseudo molecular ions peak at *m*/*z* 303.1574 [M + H]^+^ (303.1570; calcd for C_16_H_15_O_6_) and *m*/*z* 301.0715 [M − H]^−^ (301.0712; calcd for C_16_H_13_O_6_), consistent with the molecular formula C_16_H_14_O_6_ for compound **1**. The other prominent fragments in the mass spectrum were observed at *m*/*z* 285 [M − OH]^+^, 284 [M − H_2_O]^+^, 209 [M − PhOH]^+^, 192 [M − PhOH − OH]^+^, 191 [M − PhOH − H_2_O]^+^, 181, 111, and 69, characteristics of radicinol derivatives with an additional aromatic ring in the molecule.

**Figure 2:**
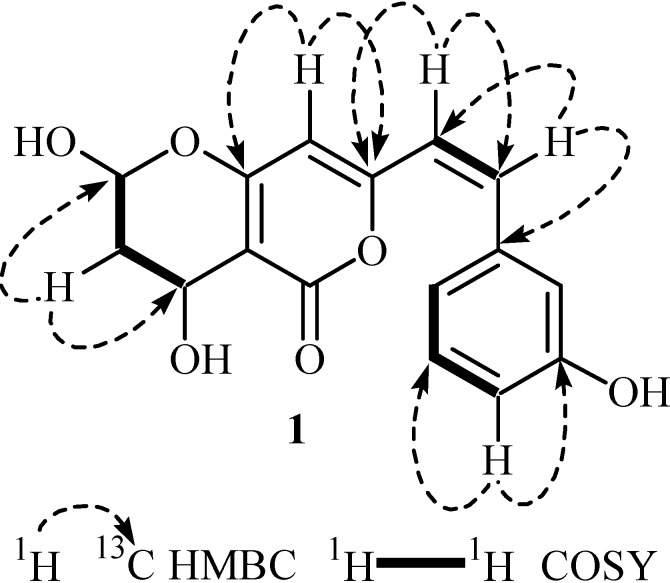
Key heteronuclear multiple bond correlation (HMBC) and important correlation spectroscopy (COSY) interactions in compound **1**.

The ^1^H-NMR spectrum of **1** displayed signals in the aromatic region between δ 6.04 to 7.98 ppm, whereas the aliphatic signals appeared between δ 3.01 to 4.95 ppm. The values of chemical shift and coupling constants of the four aromatic resonances at δ 7.98 (1H, d, *J* = 1.8 Hz, H-12), 6.74 (1H, d, *J* = 7.6 Hz, H-14), 7.24 (1H, t, *J* = 7.6 Hz, H-15), and 6.82 (1H, d, *J* = 7.8 Hz, H-16) indicated the presence of a *meta* di-substituted benzene ring. The olefinic moiety in conjugation with the benzene ring was evident by the presence of two equally split doublets at δ 4.95 and 7.18 (1H each, d, *J* = 14.1 Hz, H-9/H-10). A one proton singlet at δ 6.03 was assigned to H-7, and consistent with the ^1^H-NMR, the ^13^C-NMR data of **1** also displayed the methine type resonances at δ 96.3 (C-7), 126.3 (C-9), 129.8 (C-10), 110.6 (C-12/14), 127.9 (C-15), and 121.4 (C-16). The downfield resonance at δ 170.4 was assigned to the conjugated ester C-1. The ^13^C-NMR and DEPT experiments also revealed the presence of three quaternary signals for ring B at δ 88.2 (C-2), 169.0 (C-6), and 162.3 (C-8) and two quaternary signals for ring C at δ 137.3 (C-11) and 165.0 (C-13). The ^1^H-NMR spectrum also showed the aliphatic resonances at δ 6.04 (1H, dd, *J* = 3.4/6.7, H-5), 4.34 (1H, br s, H-3), 3.15 (1H, m, H-4a), and 3.01 (1H, m, H-4b), which were assigned to two oxy-methine and a methylene group of the dihydropyran ring A. These assignments were also confirmed by the signals in ^13^C-NMR spectrum at δ 108.5 (C-5), 58.4 (C-3), and 35.3 (C-4). The attachment of various functional groups was assigned on the basis of HMBC interactions (Figure 2) of H-4 and C-3/C-5, H-7 and C-6/C-8, H-9 and C-8/C-10, H-10 and C-9/C-11, and H-14 and C-13/C-15. The substitution pattern of **1** was thus established and the structure was further confirmed through correlations observed in COSY and HMQC experiments. The relative stereochemistry was deduced on the basis of *J* values and ^1^H-^1^H NOESY interactions of H-3 to H-5. Based on these spectral discussions, compound **1** was assigned as 3-O-acetyl-6,7,2′-trihydroxy-5,8-dimethoxyflavanone, named bipolarisenol (**1**) after the producing organism, *Bipolaris sorokiniana*.

### Enzyme Inhibition and Anti-Lipid Peroxidation Activities

After characterization of bipolarisenol through advance chromatographic and spectroscopic techniques, it was assessed for its potential in urease and acetyl cholinesterase enzymes inhibition. Urease (urea amidohydrolases, EC 3.5.1.5) is a thiol-rich and nickel-dependent metalloenzyme that can catalyze the hydrolysis of urea, thereby producing ammonia and carbamate [16]. Urease can be synthesized by numerous organisms, including plants, bacteria, algae, fungi, and invertebrates, and it also occurs in soils as a soil enzyme [17]. Importantly, ureolytic activity of bacteria, such as *Proteus mirabilis*, *Klebsiella pneumoniae*, *Staphylococcus* spp., *Salmonella* sp., and *Ureaplasma urealyticum*, is a vital virulence factor implicated in the pathogenesis of many clinical conditions, including pyelonephritis, hepatic coma, peptic ulceration, and formation of infection-induced urinary stones [18,19]. 

**Figure 3:**
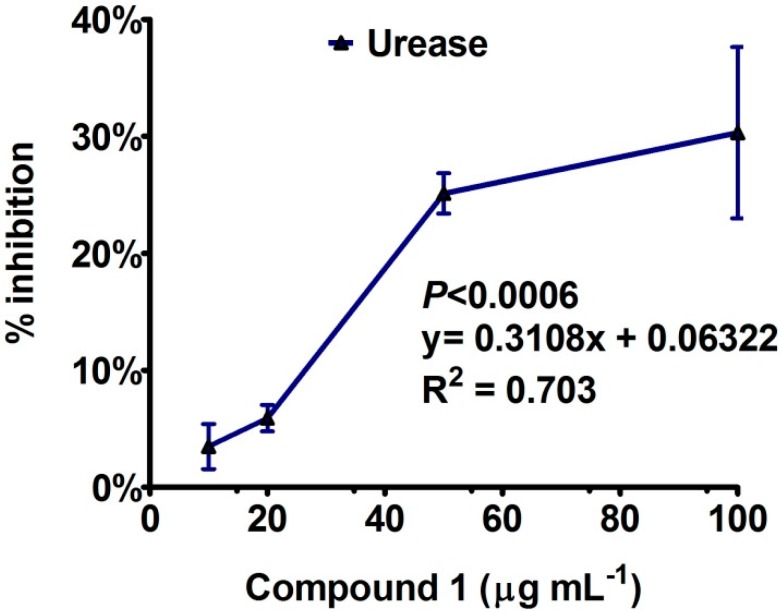
Urease inhibition activity of compound **1**, isolated from endophytic fungus. The graphs showing values of three replication with standard error.

To explore remedies of such health problems, bipolarisenol was assessed using three different concentrations, while the inhibition response against each dose was analyzed by linear regression of dose-dependent enzyme inhibition curve fitting and presented in curve-fit graph (GraphPad prism 5.05, GraphPad Software, Inc., La Jolla, CA, USA). The inhibition pattern is given in detail in Figure 3. The results showed that compound **1** exhibited a dose-dependent response with *R*^2^ values ranging above 70%. A dose of 10 to 100 µg·mL^−1^ revealed moderate suppression of urease enzyme with IC_50_ (81.62 ± 4.61 µg·mL^−1^) value suggesting that higher concentrations might help to completely hydrolyze the enzymatic activity. In continuation of previous work, natural products with flavonoids skeleton (quercetin glycosides) or their derivatives are possessing efficient inhibition potential against urease enzyme [20,21,22].

**Figure 4:**
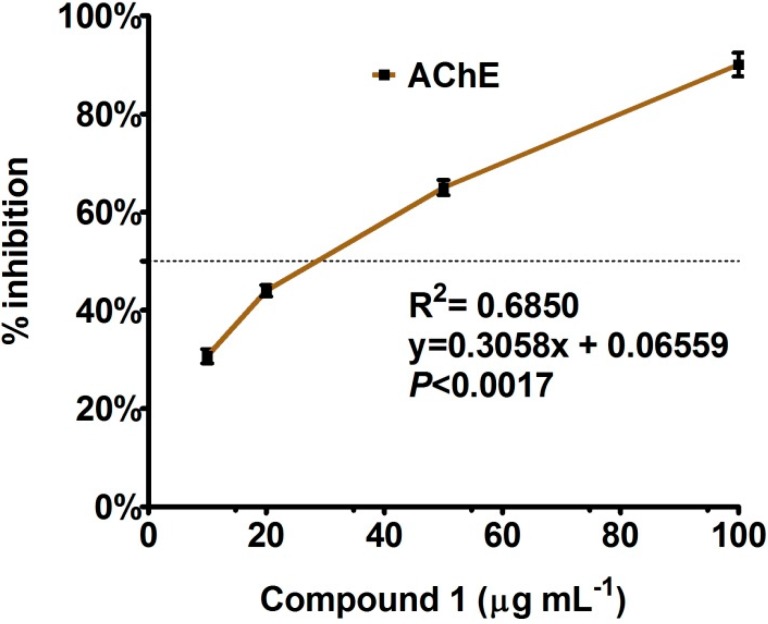
Acetyl cholinesterase inhibition activity of compound **1**, isolated from endophytic fungus. The graph showing values of three replications with standard error.

In case of acetyl cholinesterase inhibition, bipolarisenol was assayed, which showed a higher *R*^2^ values for the curve-fit against 10 to 100 µg·mL^−1^ (Figure 4). The response was dose-dependent. The IC_50_ value was low 67.23 ± 5.12 µg·mL^−1^. In response to 50 and 100 µg·mL^−1^, the AChE inhibition curve was sharp with 67 and 88% of inhibition. AChE inhibition has been emerged as a major therapeutic target [23]. AD—galantamine, rivastigmine and donepezil are acetylcholinesterase inhibitors apart from the NMDA antagonist memantine [24]. Natural products have already proven to be promising sources of useful acetylcholinesterase (AChE) inhibitors [25,26]. Compound 1 could be a promising for similar potential.

**Figure 5:**
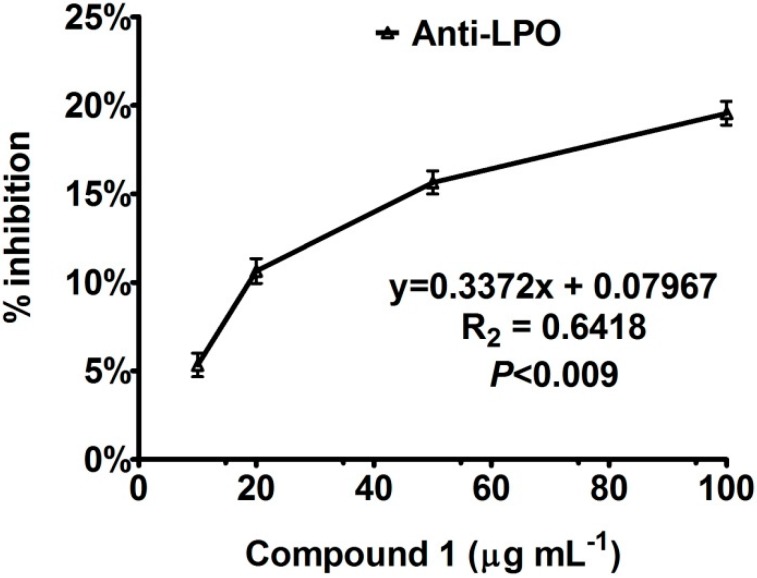
Anti-lipid peroxidation activity of compound **1**, isolated from endophytic fungus. The graph showing values of three replications with standard error.

In addition to urease and AChE, we also assessed the potential of bipolarisenol for its anti-lipid peroxidation potential. Lipid peroxidation, a well-established mechanism of cellular injury in plants and animals, is used as an indicator of oxidative stress in cell wall and tissues. Lipid peroxides are unstable and decompose to form a complex series of compounds including reactive carbonyl compounds. Polyunsaturated fatty acid peroxides generate malondialdehyde (MDA), as byproduct of lipid disintegration. The results showed that bipolarisenol extended a dose-dependent response towards anti-lipid peroxidation (Figure 5). The new compound also showed a moderate anti-lipid peroxidation potential (IC_50_ = 168.91 ± 4.23 µg·mL^−1^).

## 3. Experimental Section 

### 3.1. Endophyte Isolation and Identification

The leaves of *Rhazya stricta*, growing in the wild mountains of Jabal Al-Akhdar (23°04′22.00′′ N; 57°40′07.00′′ E), Sultanate of Oman, were detached from the plant. About 20 leaf samples were randomly collected from different trees and were stored in sterilized polythene zip-bag during transportation to the lab. The leaf samples were surface sterilized using the method of Arnold *et al.* [27]. Briefly, the samples sterilized with 5% sodium hypochlorite (30 min in a shaking incubator at 120 rpm) and washed with autoclaved deionized distilled water (DDW) to remove surface contaminants microorganisms. The tissues (1 mm) were placed on Hagem media (glucose, 0.5%; KH_2_PO_4_, 0.05%; MgSO_4_·7H_2_O, 0.05%; NH_4_Cl 0.05%; FeCl_3_, 0.1%; streptomycin, 80 ppm; and agar, 1.5%; pH 5.8 ± 0.2) to isolate the newly emerging fungal spots. The isolated new fungus (LK12) was further grown on potato dextrose agar (PDA) and Czapek broth (4.5 liters; glucose, 1%; peptone, 1%; KCl, 0.05%; MgSO_4_·7H_2_O, 0.05%; and FeSO_4_·7H_2_O, 0.001%; pH 7.3 ± 0.2; grown for 21 days at shaking incubator, 120 rpm; and temperature, 28 °C). The culture was separated through centrifugation (5000*× g* at 4 °C for 10 min) to obtain culture medium (culture filtrate (CF), 4.5 L) and fungal mycelia (120.4 g), which was lyophilized (Virtis Freeze Dryer, Gardiner, NY, USA) for molecular identification. 

Genomic DNA from the fungal mycelia was extracted according to the method of Khan *et al.* [28]. Fungal endophyte was identified by sequencing the internal transcribed regions (ITS) using universal primers: ITS-1; 5′-TCC GTA GGT GAA CCT GCG G-3′ and ITS-4; 5′-TCC TCC GCT TAT TGA TAT GC-3′. The BLAST search program was used to compare the sequence homology of similar nucleotide sequences of ITS region. The closely related sequences obtained were aligned through CLUSTAL W using MEGA version 6.06, software [29], and the maximum parsimony tree was constructed using the same software. The bootstrap replications (1K) were used as a statistical support for the nodes in the phylogenetic tree.

### 3.2. Secondary Metabolite Structure Elucidation

Optical rotations were measured on a JASCO DIP 360 polarimeter. IR spectra were recorded on a Bruker, ATR-Tensor 37 spectrophotometer. ESI mass spectra were recorded on QSTAR XL (Applied Biosystem). The capillary voltage was maintained between 5 and 5.5 kV. The ^1^H- and ^13^C-NMR spectra were recorded on Bruker NMR spectrometers operating at 600 MHz (150 MHz for ^13^C). The chemical shifts values are reported in ppm (δ) units and the coupling constants (J) are given in Hz. Data are reported as follows: chemical shift (multiplicity (singlet (s), doublet (d), triplet (t), quartet (q) and multiplet (m)), coupling constants (Hz), and integration). Minor compounds were purified by using recycling preparative High Performance Liquid Chromatography (HPLC) by JAI using silica gel column and 7:3 ethyl acetate/*n*-hexane systems. For TLC, pre coated aluminum sheets (silica gel 60F-254, E. Merck, Darmstadt, Germany) were used. Visualizations of the TLC plates were achieved under the UV light at 254 and 366 nm, and also by spraying with the ceric sulfate and ninhydrin reagent.

### 3.3. Extraction and Isolation

The mycelial mats and the culture filtrate were extracted completely with ethyl acetate (EtOAc). Both the extracts were compiled and dried over sodium sulfate (anhydrous) and concentrated *in vacuo* to afford the crude extract (2.1 g). The EtOAc extract was then subjected to silica gel column chromatography using gradients of ethyl acetate/*n*-hexane system to afford five fractions (Fr.A to Fr.E). Fr.E was further subjected to recycling preparative HPLC (JAI) analysis. Compound **1** (5.1 mg) was purified at a retention time of 23 min by using ethyl acetate/*n*-hexane (6:4) in a silica gel column with the flow rate 3.5 mL/min after five recycles.

*Bipolarisenol* (**1**): colorless amorphous powder. ${\left[\alpha \right]}_{D}^{30}$ = −24.2 (*c* = 0.6, CH_3_OH). UV λ_max_ (CH_3_OH): 340, 264 nm. IR *ν*_max_ (CH_3_OH): 3595, 1695, and 1590 cm^−1^. ^1^H-NMR (600 MHz, DMSO-*d_6_*), δ 7.98 (1H, d, *J* = 1.8 Hz, H-12), 7.24 (1H, t, *J* = 7.6 Hz, H-15), 7.18 (1H, d, *J* = 14.1 Hz, H-10), 6.82 (1H, d, *J* = 7.8 Hz, H-16), 6.74 (1H, d, *J* = 7.6 Hz, H-14), 6.04 (1H, dd, *J* = 3.4/6.7 Hz, H-5), 6.03 (1H, s, H-7), 4.95 (1H, d, *J* = 14.1 Hz, H-9), 4.34 (1H, br s, H-3), 3.15 (1H, m, H-4), 3.01 (1H, m, H-4). ^13^C-NMR (150 MHz, DMSO-*d_6_*), δ 170.4 (C-1), 169.0 (C-6), 165.0 (C-13), 162.3 (C-8), 137.3 (C-11), 129.8 (C-10), 127.9 (C-15), 126.3 (C-9), 121.4 (C-16), 110.6 (C-12/14), 108.5 (C-5), 96.3 (C-7), 88.2 (C-2), 58.4 (C-3), 35.3 (C-4). EI-MS (70 eV): *m*/*z* 302 [M]^+^, 285 [M − OH]^+^, 284 [M − H_2_O]^+^, 209 [M − PhOH]^+^, 192 [M − PhOH − OH]^+^, 191 [M − PhOH − H_2_O]^+^, 181, 111, 69. HR-ESI-MS: 303.1574 [M + H]^+^ (303.1570; calculated for C_16_H_15_O_6_), 301.0715 [M − H]^−^ (301.0712; calculated for C_16_H_13_O_6_).

### 3.4. Anti-Lipid Peroxidation and Enzyme Inhibition Assays

The potential of compounds to inhibit the extent of lipid peroxidation was assessed through a modified method of thiobarbituric acid reactive substances (TBARS) [30]. This was based on the peroxidation of a liposome (phosphatidyl-choline 50 mg/mL) induced by iron chloride (200 µL; 1 mM) containing potassium chloride (300 mM) in the presence of sample (50 µL). Peroxidation was initiated by ascorbate (125 µL with 0.16 mM) and the reaction mixture was incubated for 30 min at 37 °C. A mixture of trichloroacetic acid (0.75 mL with 1.5:1 (*v*:*v*)) and TBA (0.38%) was added to the reaction mixture. It was kept in boiling water for 30 min until the pink color appears, which was measured on ELISA at A_535_. A control without compound was used as negative control while butyl hydroxy toluene (BHT) was used as positive control. The inhibition (IP% = (1 − At/Ao) × 100); where At and Ao are compound and control absorbance after incubation for 30 min. The experiment was repeated three times. 

Urease enzyme inhibition activities of the compound were performed according to the method of Golbabaei *et al.* [31]. Briefly, a 25 μL solution of Jack bean Urease, 55 μL of 100 mM urea dissolved in phosphate buffer (0.01 M K_2_HPO_4_·3H_2_O, 1.0 mM EDTA and 0.01 M LiCl_2_; pH 8.2) and different concentrations of the compounds (10, 20, 50 and 100 µg/mL). The reaction mixture was incubated at 30 °C for 15 min in 96-well plate. The production of ammonia was measured by indophenol method to determine the urease inhibitory activity. The phenol reagent (45 μL, 1% *w*/*v* phenol and 0.005% *w*/*v* sodium nitroprusside) and alkali reagent (70 μL, 0.5% *w*/*v* sodium hydroxide and 0.1% NaOCl) were added to each well and the increasing absorbance at 630 nm was measured after 50 min, using an ELISA microplate reader. The change in absorbance per minute was noted. All the tests were performed in triplicate. The percentage inhibition was calculated from the following equation. Inhibitory activity (%) = 100 − (ODtest well/ODcontrol) × 100. Thiourea was used as the standard inhibitor with 92% ± 1.50% Inhibition.

Acetyl Cholinestrase (AChE) inhibitory activity of the compound was measured by using method of Ingkaninan *et al.* [32]. This method is based on the enzyme hydrolyses of substrate acetylthiocholine iodide (ATCI; 15 mM) resulting in the product thiocholine which reacts with Ellman’s reagent 5,5′-dithiobis [2-nitrobenzoic acid] (DTNB; 3 mM) to produce 2-nitrobenzoate-5-mercaptothiocholine and 5-thio-2-nitrobenzoate, which can be detected at 412 nm. Tris–HCl (50 mM; pH 8.0) was used as a buffer. AChE used in the assay was from electric eel (type VI-S lyophilized powder, 518 U/mg solid, and 844 U/mg protein). The enzyme stock solution (518 U/mL) was kept at −80 °C. The further enzyme-dilution was done in 0.1% BSA in buffer. DTNB was dissolved in Tris buffer (0.1 M NaCl and 0.02 M MgCl_2_). ATCI was dissolved in deionized water. 

In the 96-well plates with 100 µL DTNB, 20 µL of 0.26 U/mL AChE, 40 µL buffer (50 mM Tris pH 8.0), and 20 µL sample in various concentrations dissolved in buffer containing not more than 10% methanol were added to wells. After mixing, the plate was incubated for 15 min (25 °C) and then the absorbance was measured at 412 nm in xMark ELISA reader (BioRad, Berkeley, CA, USA). The blank was used without compound in the reaction mixture. The enzymatic reaction was initiated by the addition of 20 µL of ATCI and the hydrolysis of acetylthiocholine was monitored by reading the absorbance every 5 min for 20 min. Galanthamine was used as positive control. All the reactions were performed in triplicate. The percentage inhibition was calculated as follows: Inhibition (%) = ES/E × 100, where, E is the activity of the enzyme without extract and S is the activity of enzyme with the extract. 

### 3.5. Statistical Analysis

The data shown are the mean values of at least three replicate experiments and expressed as means ± SD. Statistical analyses were conducted using GraphPad Prism software 5.03 package.

## 4. Conclusions

Endophytic fungus, *Bipolaris sorokiniana* LK12, which was isolated from the leaves of alkaloid rich *Rhazya stricta*, produced a new compound, bipolarisenol. It is the first ever report from this fungus. Furthermore, bipolarisenol was assessed for its potential role in enzyme inhibition of urease and acetyl cholinesterase (AChE), where it showed significantly higher inhibition of AChE activity as compared to urease and anti-lipid peroxidation. The results of the present study may lead to the conclusion that endophytes are considered potential source for novel bioactive products. Thus, the endophytic fungi play an important role in the search for natural compounds as an alternative source for the production of therapeutic agents and bioactive metabolites that are not easily synthesized and have a high activity against pathogenic microorganisms. However, the present study will serve only as a prelude to the more comprehensive studies on the chemistry and biology of the bioactive natural products produced by these endophytes.
